# S-Nitrosylation of RhoGAP Myosin9A Is Altered in Advanced Diabetic Kidney Disease

**DOI:** 10.3389/fmed.2021.679518

**Published:** 2021-07-14

**Authors:** Qi Li, Delma Veron, Alda Tufro

**Affiliations:** ^1^Department of Pediatrics/Nephrology, New Haven, CT, United States; ^2^Department of Cell and Molecular Physiology, Yale School of Medicine, New Haven, CT, United States

**Keywords:** diabetic kidney disease, cell cross-talk, RhoA, S-nitrosylation, *MYO9A*, actin

## Abstract

The molecular pathogenesis of diabetic kidney disease progression is complex and remains unresolved. Rho-GAP *MYO9A* was recently identified as a novel podocyte protein and a candidate gene for monogenic FSGS. Myo9A involvement in diabetic kidney disease has been suggested. Here, we examined the effect of diabetic milieu on Myo9A expression *in vivo* and *in vitro*. We determined that Myo9A undergoes S-nitrosylation, a post-translational modification dependent on nitric oxide (NO) availability. Diabetic mice with nodular glomerulosclerosis and severe proteinuria associated with doxycycline-induced, podocyte-specific *VEGF*_164_ gain-of-function showed markedly decreased glomerular Myo9A expression and S-nitrosylation, as compared to uninduced diabetic mice. Immortalized mouse podocytes exposed to high glucose revealed decreased *Myo9A* expression, assessed by qPCR, immunoblot and immunocytochemistry, and reduced Myo9A S-nitrosylation (SNO-Myo9A), assessed by proximity link assay and biotin switch test, functionally resulting in abnormal podocyte migration. These defects were abrogated by exposure to a NO donor and were not due to hyperosmolarity. Our data demonstrate that high-glucose induced decrease of both *Myo9A* expression and SNO-Myo9A is regulated by NO availability. We detected S-nitrosylation of Myo9A interacting proteins RhoA and actin, which was also altered by high glucose and NO dependent. RhoA activity inversely related to SNO-RhoA. Collectively, data suggest that dysregulation of SNO-Myo9A, SNO-RhoA and SNO-actin may contribute to the pathogenesis of advanced diabetic kidney disease and may be amenable to therapeutic targeting.

## Introduction

Diabetic kidney disease (DKD) is a major complication of both type 1 and type 2 diabetes that leads to renal failure, and the single most frequent cause of end-stage renal disease (ESRD) worldwide (1). In the last few years novel therapies led to remarkable improvement of metabolic control in diabetic patients (2, 3). However, the prevalence and progression of DKD have not decreased as yet (2, 3). Incomplete understanding of the molecular mechanisms involved in DKD progression has precluded the development of effective treatments to prevent, halt or reverse progression to ESRD (4–6). In an effort to address this, we investigated the role of a novel podocyte protein, Myosin 9A, in progression to advanced DKD.

*MYO9A* was recently identified as a novel candidate gene for monogenic FSGS (7). This study also suggested dysregulation of *Myo9A* expression in other experimental proteinuric diseases, such as nephrotic syndrome and diabetic nephropathy (7). The relevance of *Myo9A* involvement in diabetic kidney disease is presently unknown. Myosins are a super family of actin binding molecular motors that regulate cell shape and motility, organelle trafficking and signaling (8–10). Several non-muscle myosins regulate foot process actin dynamics in podocytes (11). The 40 gene members of the myosin family share a common structure consisting of head, neck and tail domains and have been grouped in 18 classes based on their distinctive features. Class 2 non-muscle myosin *MYH9* has been implicated in the pathogenesis of DKD (12) and MYO1E mutations cause monogenic FSGS (13). Class 9 myosins' unique features are their RhoGTPase-activating protein (Rho-GAP) tail domain and a loop insert in their head domain (14). Myosin 9A (Myo9A) crosslinks and bundles actin, inactivates RhoA and controls epithelial cell junction assembly (9, 10, 14). *Myo9A* is expressed by epithelial cells in brain, kidney, testis and lung (15). In the kidney Myo9A localizes to podocytes and proximal tubular cells (7). *Myo9A* loss-of-function increases kidney RhoA activity and alters podocyte function (7).

In diabetes, hyperglycemia induces uncoupling of nitric oxide synthase homodimers and overproduction of reactive oxygen species (ROS) relative to antioxidant molecules, resulting in low nitric oxide (NO) availability (16–18). NO signals through two distinct pathways: activation of guanylyl cyclase to produce cyclic GMP (cGMP) and protein S-nitrosylation. S-nitrosylation is the reversible, oxidative addition of NO to Cys residues to form S-nitrosothiols (SNOs) that modifies myriad proteins, providing a redox-based cellular signaling mechanism that conveys the ubiquitous influence of NO on cellular function (19). S-nitrosylation regulates protein activity of multiple proteins that play important roles in DKD, including all nitric oxide synthase (NOS) isoforms, guanylyl cyclase (GC), hypoxia-inducible factor1α (HiF1α), thioredoxin (20–23), as well as in cytoskeletal dynamics, such as actin and RhoA (24–26).

Since Myo9A directly interacts with both actin and RhoA (27, 28), we hypothesized that Myo9A might undergo S-nitrosylation and thereby participate in a transnitrosylation cascade to serve NO signaling. The goals of this study were to determine whether Myo9A dysregulation is involved in the severity of DKD and to assess whether the molecular mechanism involves Myo9A S-nitrosylation. We documented that Myo9A is S-nitrosylated *in vivo* and in cultured podocytes in control conditions. Diabetic mice with advanced DKD revealed downregulation of Myo9A expression and S-nitrosylation. Cultured podocytes showed that high glucose-induced Myo9A dysregulation is NO dependent and involves actin and RhoA S-nitrosylation. These findings uncover Myo9A relevance in advanced DKD and identify a targetable pathway that might influence DKD progression involving cross-talk among multiple nephron cell types.

## Materials and Methods

### Animal Model

Experiments were performed using kidney tissue from *podocin*-rtTA:tet-O-*VEGF*_164_ (i*VEGF*) diabetic mice, herein called DM-i*VEGF* mice, previously reported (29). *Podocin*-*rtTA:tet-O*-*VEGF*_164_ are podocyte-specific inducible transgenic mice that overexpress VEGF_164_ in podocytes upon induction with doxycycline, as described (30). Mice were crossbred on FVB/N background. Diabetes was induced using streptozotocin (50 mg/kg body weight i.p. for 5 consecutive days) following the Animal Models of Diabetic Complications Consortium (www.AMDCC.org) short protocol in 5.0 ± 0.6 week old i*VEGF* mice (*n* = 15). Diabetic i*VEGF* mice (DM-i*VEGF*) were fed doxycycline containing chow (0.625 mg/g chow; Harlan-Teklad) (DM-i*VEGF* +dox, *n* = 8), or fed standard chow (DM-i*VEGF*-dox, *n* = 7) for 12 weeks to induce *VEGF*_164_ expression or serve as diabetic controls, respectively (29). At the end of 12 weeks, mice were anesthetized and kidneys were perfused with sterile PBS and excised prior to euthanasia. All experimental protocols were approved by the Institutional Animal Care and Use Committee at Yale University School of Medicine.

### Cell Culture

Immortalized mouse podocytes were cultured in RPMI-1640 medium (11875-093, Life Technologies), 1% Insulin-Transferrin-Selenium (41400-045, Life Technologies), 10% heat inactivated FBS (10438-026, Life Technologies), 1% Pen/Strep at 33°C with 5% CO_2._ Podocyte differentiation was induced by incubation at 37°C for 7 days. Podocytes incubated in control medium (11 mM D-glucose), medium + 25 mM glucose, medium + 25 mM mannitol, or medium + 25 mM glucose + 10μM DETA NONOate (#82120,Cayman Chemical) for 24 h. For immunocytochemistry and proximity link assays, podocytes were cultured in 4-chamber slides; for cell migration assays, podocytes (1 × 10^5^cell/ml) were cultured in 35 mm dishes.

### Immunoblot/Immunoprecipitation

Kidneys were snap frozen in liquid nitrogen at the time of euthanasia, and podocytes were pelleted by centrifugation at the end of culture experiments. Both tissues and cells were lyzed in lysis buffer (1% NP-40, 1% Triton X, 50 mM Hepes, 150 mM NaCl, 0.1 mM EDTA, 0.1 mM Neocuproine, complete protease inhibitor, Roche) for immunoblot and co-immunoprecipitation analysis, as previously described (7, 31). Proteins were resolved by SDS-PAGE in 10% or 4–20% SDS–polyacrylamide gels (BioRad), transferred to polyvinylidene difluoride membranes, blocked with 5% dry-milk or 5% BSA in TBST and incubated with primary antibodies: actin (A2066, Sigma), Myo9A (Abnova, clone 4C11) and RhoA (67B9,Cell Signaling), followed by appropriate species specific HRP-conjugated secondary antibodies (Jackson Immuno Research Laboratories Inc.). Immunoblotted proteins were visualized with ECL. Co-immunoprecipitation was performed using podocyte lysates, as previously described (7). Briefly, following pre-clearing with prewashed protein A agarose beads, lysate supernatants were incubated anti-MYO9A rabbit polyclonal antibody (A305-702A-M, Bethyl) at 4°C, pre-washed agarose beads were added and incubated overnight. Agarose beads were washed with PBS+protease inhibitors (Roche), spun and resuspended in Laemmli sample buffer for western blot analysis as described above.

### Histology/Immunohistochemistry/ Immunocytochemistry

Kidneys were perfused with sterile PBS for morphologic studies prior to euthanasia, incubated in 18% sucrose, embedded in optimal cutting temperature medium (OCT, Sakura Finetek USA), frozen in isopentane/dry ice and kept at −80°C for immunohistochemistry (IHC), as described (29) or processed for light microscopy. Histology was evaluated by periodic acid–Schiff's reagent (PAS) stain. Kidney frozen sections and podocytes were fixed in 4% PFA, permeabilized with 0.3% triton-X, blocked with 10% donkey serum, 5% BSA in PBST at room temperature, and incubated overnight at 4°C in primary antibodies: S-nitrosocysteine mouse monoclonal antibody (AG Scientific,1:100) and rabbit anti Myo9A (NBP1-92160, Novus, 1:50). Sections were washed, incubated with fluorescent-tagged secondary antibodies: goat anti-mouse Alexa Fluor 594 or goat anti-rabbit Alexa Fluor 488 (Life Technologies, 1:150) at room temperature. Coverslips were mounted with Vectashield + DAPI (Vector Labs). Stained sections and cells were examined using an Olympus IX 71 inverted fluorescence/phase and bright field microscope (Olympus, Tokyo, Japan) equipped with an Optronics (Goleta, CA) Microfire camera and Pictureframe version 3.00.30 software. Images were processed with Adobe Photoshop CC 2018 (Adobe Systems).

### *In situ* Proximity Ligation Assay (PLA)

Myo9A S-nitrosylation was detected and localized using an *in situ* proximity link assay, as previously described (32, 33). Here, we used Myo9A rabbit polyclonal antibody (Novus) and S-nitrosocysteine mouse monoclonal antibody (AG Scientific), Duolink PLA probes and fluorescent labeled oligonucleotides to visualize the amplified reaction product attached to the antibody protein complex, following the Duolink^®^ PLA fluorescence protocol (Sigma). Briefly, kidney frozen sections or podocytes were fixed, permeabilized and blocked as described above + 0.3% hydrogen peroxide in PBS and incubated overnight with primary antibodies Myo9A and S-nitrosocysteine at 4°C. Secondary antibodies (PLA probes) donkey anti-rabbit and anti-mouse conjugated with oligonucleotides were added and incubated at 37°C for 60 min. Sections were washed with PBS, incubated with ligation solution containing oligonucleotides for 30 min at 37°C. Ligation of oligonucleotides generates a circular DNA strand that serves as a template only if the probes are in close proximity. Then, sections were incubated at 37°C with DNA polymerase and fluorescently labeled oligonucleotides for 100 min. The amplification reaction product attaches to the antibody protein complex and is visualized as a fluorescent signal resulting from the hybridization of fluorescently labeled oligonucleotides. Kidney sections and podocytes were washed, and coverslips placed using mounting medium with DAPI (Vector). Cy3 and DAPI fluorescence signals were detected by inverted fluorescence microscopy at × 400 magnification and processed as described above.

### Biotin Switch Assay (BST)

S-nitrosylation of Myo9A, RhoA and actin was measured using a biotin switch assay (34) (S-nitrosylated protein detection kit, Cayman Chemical Co.), following the manufacturer's instructions. Briefly, podocyte lysates (1,000 μg) were re-suspended in blocking buffer to block free thiols, acetone precipitated, S-NO bonds were reduced, and the resulting free thiols were labeled with maleimide-biotin. Proteins were acetone-precipitated, the pellets were re-suspended in equal volumes of HENS/10 + 1% SDS buffer. To pull-down the biotinylated proteins we added streptavidin-agarose beads (Fluka). Beads were washed 5 times and bound proteins were eluted in 2X sample buffer. Myo9A, RhoA and actin presence in the eluates was detected by immunoblotting.

### RhoA Activity

Active RhoA was measured with Rho-activation pulldown assay (Millipore) following manufacturer's instructions. Active RhoA was detected by immunoblotting using RhoA antibody (67B9, Cell Signaling), as described (7).

### Statistical Analysis

Data are analyzed with GraphPad-Prism-8 software (San Diego, CA) using unpaired Student's-*t*-test with Welch's correction, Welch's or Brown-Forsythe ANOVA, as appropriate. Non-parametric Kriskall-Wallis and Mann-Whitney test were used to analyze RhoA activity data. *P* < 0.05 was deemed statistically significant. Data are expressed as mean ± SD, unless otherwise indicated.

## Results

### Kidney Myo9A Expression and S-Nitrosylation in Diabetic Kidney Disease

**Figure 1:**
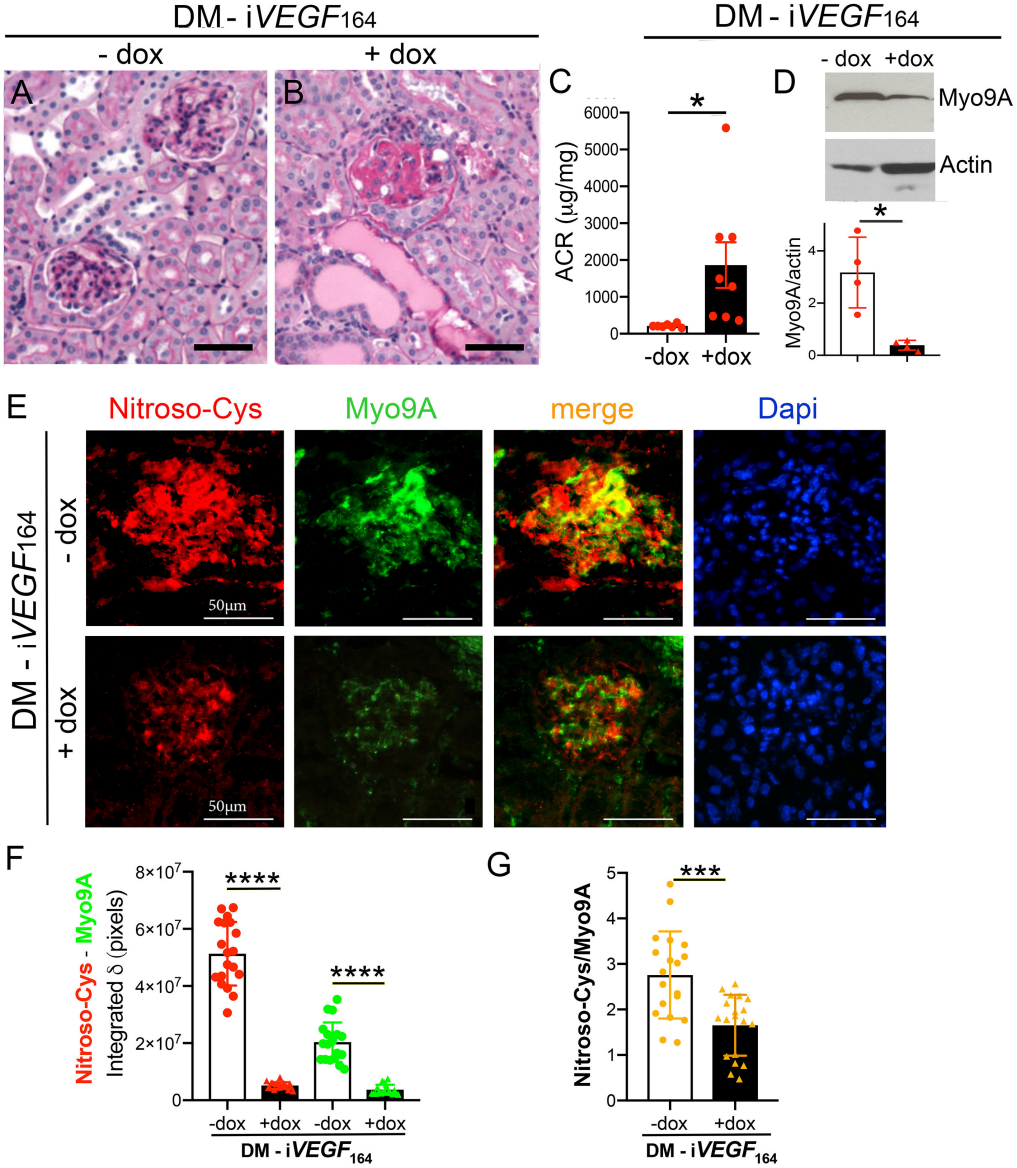
Myo9A is downregulated in advanced diabetic kidney disease. **(A)** Kidney PAS stain from uninduced diabetic mouse (DM-i*VEGF*_164_, - dox) showing mild mesangial proliferation; **(B)** kidney PAS stain from induced diabetic mouse (DM-i*VEGF*_164_, + dox) showing nodular glomerulosclerosis and large protein casts; **(C)** Urine ACR (albumin:creatinine ratio, mg/mg) shows mild albuminuria in uninduced diabetic mice (DM-i*VEGF*_164_, - dox, *n* = 7) and nephrotic range proteinuria in induced diabetic mice (DM-i*VEGF*_164_, + dox, *n* = 8), unpaired *t*-test with Welch's correction, *P* = 0.033; **(D)** representative immunoblot shows decreased kidney Myo9A expression in mice with advanced DKD (DM-i*VEGF*_164_, + dox); quantitation of Myo9A expression normalized to actin confirms significant Myo9A downregulation in *n* = 4 immunoblots (kidney lysates pooled from 4 to 6 mice/experimental group), mean ± SD, *P* < 0.05; **(E)** Fluorescence IHC shows S-nitrosylated proteins (red) and Myo9A (green) partially co-localized (merge) in glomeruli from uninduced diabetic kidneys (DM-i*VEGF*_164_, - dox), both Myo9A and nitroso-Cys IF signals are reduced in glomeruli from kidneys with advanced DKD (DM-i*VEGF*_164_, + dox); **(F)** quantitation of Myo9A and nitroso-Cys IF signals confirm a dramatic decrease in glomerular Myo9A expression and S-nitrosylated proteins in kidneys with advanced DKD (DM-i*VEGF*_164_, + dox), mean ± SD, *n* = 19 glomeruli/experimental group (each from 3 to 5 mice), unpaired *t*-test with Welch's correction, *p* < 0.0001; **(G)** quantitation of the IF signals' ratio Nitroso-Cys/Myo9A shows significant decrease in kidneys with advanced DKD, mean ± SD, *n* = 19 glomeruli/experimental group, unpaired *t*-test with Welch's correction, *p* = 0.0002. Scale bars = 50μm. *p < 0.05, ***p < 0.005, ****p < 0.0001.

To begin to understand the role of *Myo9A* in DKD progression we compared Myo9A expression in diabetic mice with mild vs. advanced diabetic kidney disease (DKD). We examined Myo9A expression and distribution of S-nitrosylated proteins in kidneys from mice with streptozotocin-mediated diabetes and doxycycline-inducible, podocyte *VEGF*_164_ overexpression (DM- i*VEGF*_164_) (29, 30). As previously reported (29), uninduced diabetic mice (− dox) show discrete glomerular changes (Figure 1A) and mild albuminuria (ACR: 212 ± 18 μg/mg creatinine, Figure 1C), whereas doxycycline-induced diabetic mice overexpressing *VEGF*_164_ (DM- i*VEGF*_164_+dox) develop severe diabetic nodular glomerulosclerosis (Figure 1B) and nephrotic range proteinuria (ACR:1947 ± 708 μg/mg creatinine, Figure 1C), herein referred to as advanced DKD. Induced and uninduced diabetic mice developed similar hyperglycemia (29). Using immunoblotting we determined that kidney Myo9A expression is significantly decreased in mice with advanced DKD (+ dox) as compared to mice with mild DKD (- dox) (Figure 1D) and non-diabetic mice (7).

Dual immunofluorescence labeling (IF) revealed that Myo9A and S-nitrosylated proteins localize to glomeruli from all diabetic mice (Figure 1E). S-nitrosylated proteins partially co-localize with Myo9A. We observed a significant decrease of glomerular Myo9A and S-nitrosylated proteins in induced diabetic mice (+ dox) as compared to non-induced diabetic mice (- dox). Quantitation of Myo9A and nitroso-Cys IF signals is shown in Figure 1F and the ratio of Nitroso-Cys/Myo9A IF signals is shown in Figure 1G. Together, IF data indicate that glomerular Myo9A and S-nitrosylated proteins partially co-localize and are decreased in the setting of advanced diabetic glomerulosclerosis, raising the possibility that Myo9A could be a S-nitrosylated protein.

**Figure 2:**
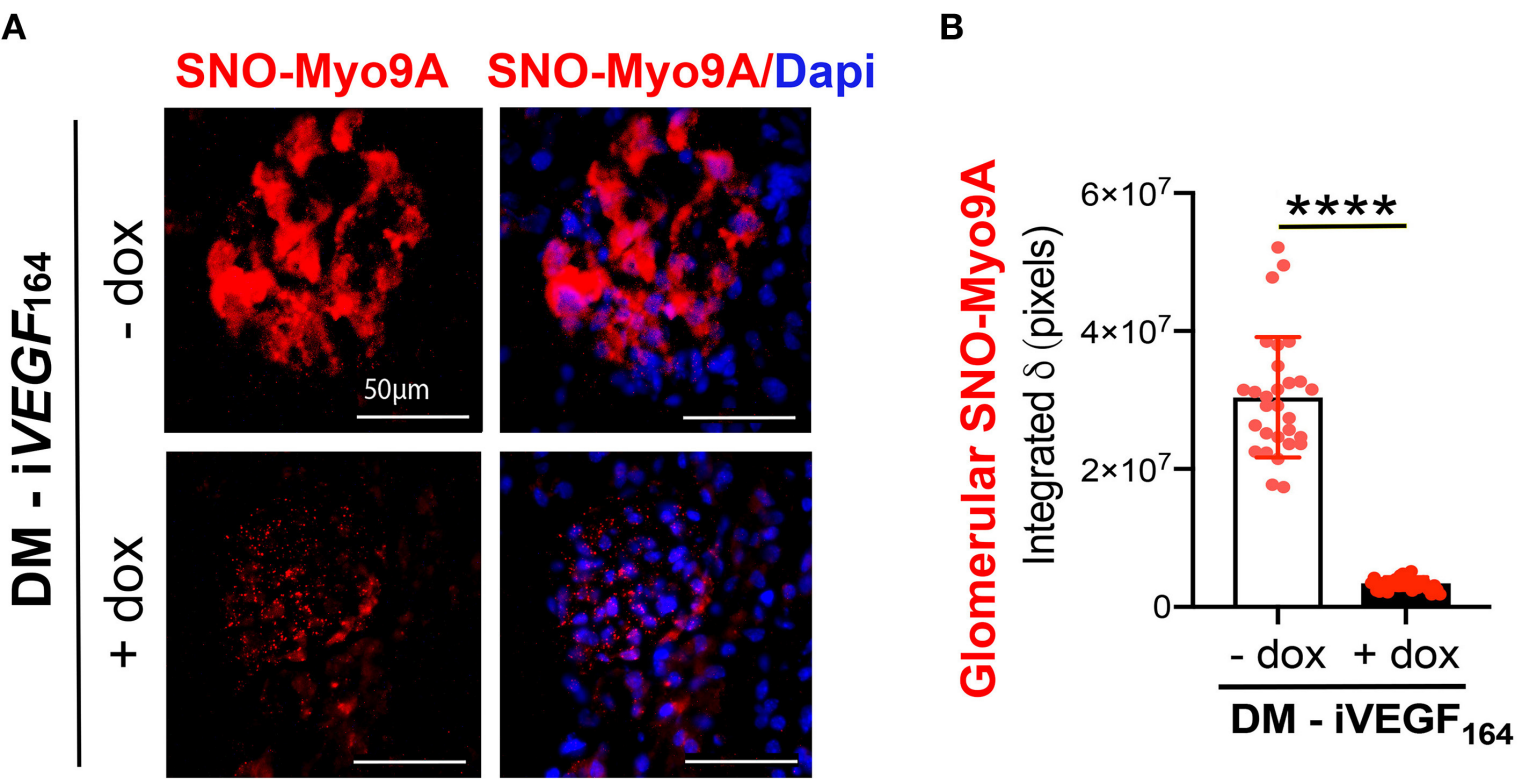
Glomerular Myo9A is S-nitrosylated in diabetic mice. **(A)** Proximity link assay IF signal (red) identifies abundant S-nitrosylated Myo9A (SNO-Myo9a) in glomeruli from uninduced diabetic mice with mild DKD (DM-i*VEGF*_164_ - dox), whereas SNO-Myo9A is clearly reduced in glomeruli from induced diabetic mice with advanced DKD (DM-i*VEGF*_164_, + dox). Dapi (blue) identifies cell nuclei. Scale bars = 50 μm. **(B)** Quantification of PLA IF signals, mean ± SD, *n* = 29–31/experimental group, unpaired *t*-test with Welch's correction, ****p < 0.0001.

To determine *in situ* whether glomerular Myo9A is S-nitrosylated we utilized a proximity link assay (PLA) (32, 33). Immunofluorescent PLA signals shown in Figure 2A demonstrate the presence of nitroso-Cys residues linked to Myo9A in uninduced glomeruli (- dox) and dramatically reduced SNO-Myo9A signal in glomeruli from induced (+ dox) diabetic mice. Quantitation of PLA signals is shown in Figure 2B. PLA data indicate that glomerular Myo9A is S-nitrosylated and that this post-translational modification is significantly downregulated in mouse kidneys with advanced DKD.

### Podocyte Myo9A S-Nitrosylation Regulation by Glucose

**Figure 3:**
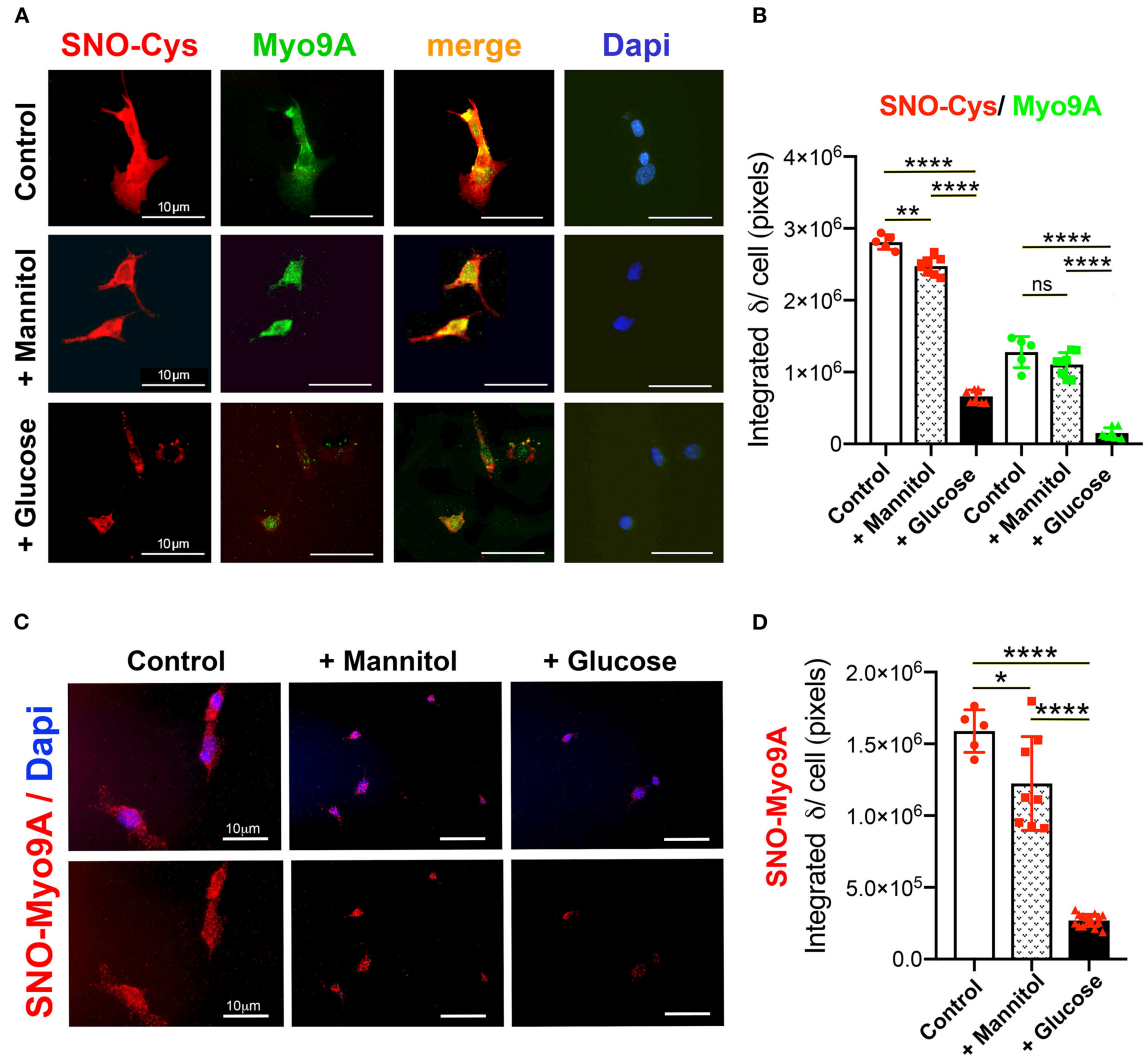
Podocyte Myo9A expression and S-nitrosylation are downregulated by high glucose. **(A)** IHC shows abundant SNO-Cys proteins (red) and Myo9A (green) partially co-localized in normal podocytes (*top panels*) and in podocytes exposed to mannitol (*middle panels*), whereas both SNO-Cys and Myo9A signals are clearly reduced in podocytes exposed to high glucose (*bottom panels*). Scale bars= 10μm. **(B)** Quantitation of IHC IF signals demonstrate highly significant decrease in Myo9A and SNO-Cys proteins in podocytes exposed to high glucose, data expressed as mean ± SD, *n* = 24–32 cells/experimental group, Welch's ANOVA *p* < 0.0001, unpaired *t*-test with Welch's correction *p* < 0.027 or n.s. control vs. mannitol, *p* < 0.0001 mannitol vs. high glucose. **(C)** Proximity link assay IF signal (red) identifies SNO-Myo9A in control podocytes, a mild decrease in podocytes exposed to mannitol and barely detected SNO-Myo9A in podocytes exposed to high glucose; Dapi (blue) identifies cell nuclei. Scale bars = 10μm. **(D)** Quantification of PLA IF signals, mean ± SD, *n* = 20–31 cells/experimental group, Welch's ANOVA *p* < 0.0001, unpaired *t*-test with Welch's correction *p* < 0.02 control vs. mannitol, *p* < 0.0001 mannitol vs. high glucose. *p < 0.05, **p < 0.01, ****p < 0.0001.

A previous study demonstrated Myo9A expression in glomerular podocytes *in vivo* and in immortalized mouse and human podocytes (7). Therefore, we examined the effect of hyperglycemia on podocyte Myo9A expression and on S-nitrosylation. Immortalized mouse podocytes were exposed to normal glucose, mannitol, or high glucose, as described in the methods section. Mannitol was used as a control for hyperosmolarity-induced changes. Using IF dual labeling, we determined that Myo9A co-localizes with nitroso-Cys (SNO-Cys) signals in undifferentiated podocytes on normal glucose medium (Figure 3A, control, *top panels*) and differentiated podocytes exposed to mannitol (Figure 3A, *middle panels*), while differentiated podocytes exposed to high glucose showed reduced Myo9A and nitroso-Cys (SNO-Cys) proteins (Figure 3A, *bottom panels*). Quantification of IF signals demonstrating these highly significant changes are shown in Figure 3B.

Proximity linked assay (PLA) revealed *in situ* S-nitrosylated Myo9A (SNO-Myo9A) fluorescent signals in podocytes grown in normal glucose and in podocytes exposed to mannitol, whereas SNO-MyoA signals were barely detected in podocytes exposed to high glucose (Figure 3C). Quantitation of PLA IF signals confirmed that exposure to high glucose significantly decreases SNO-Myo9A in podocytes (Figure 3D), demonstrating that SNO-Myo9A is regulated by glucose in podocytes.

### Podocyte Myo9A S-Nitrosylation Regulation by Nitric Oxide Availability

**Figure 4:**
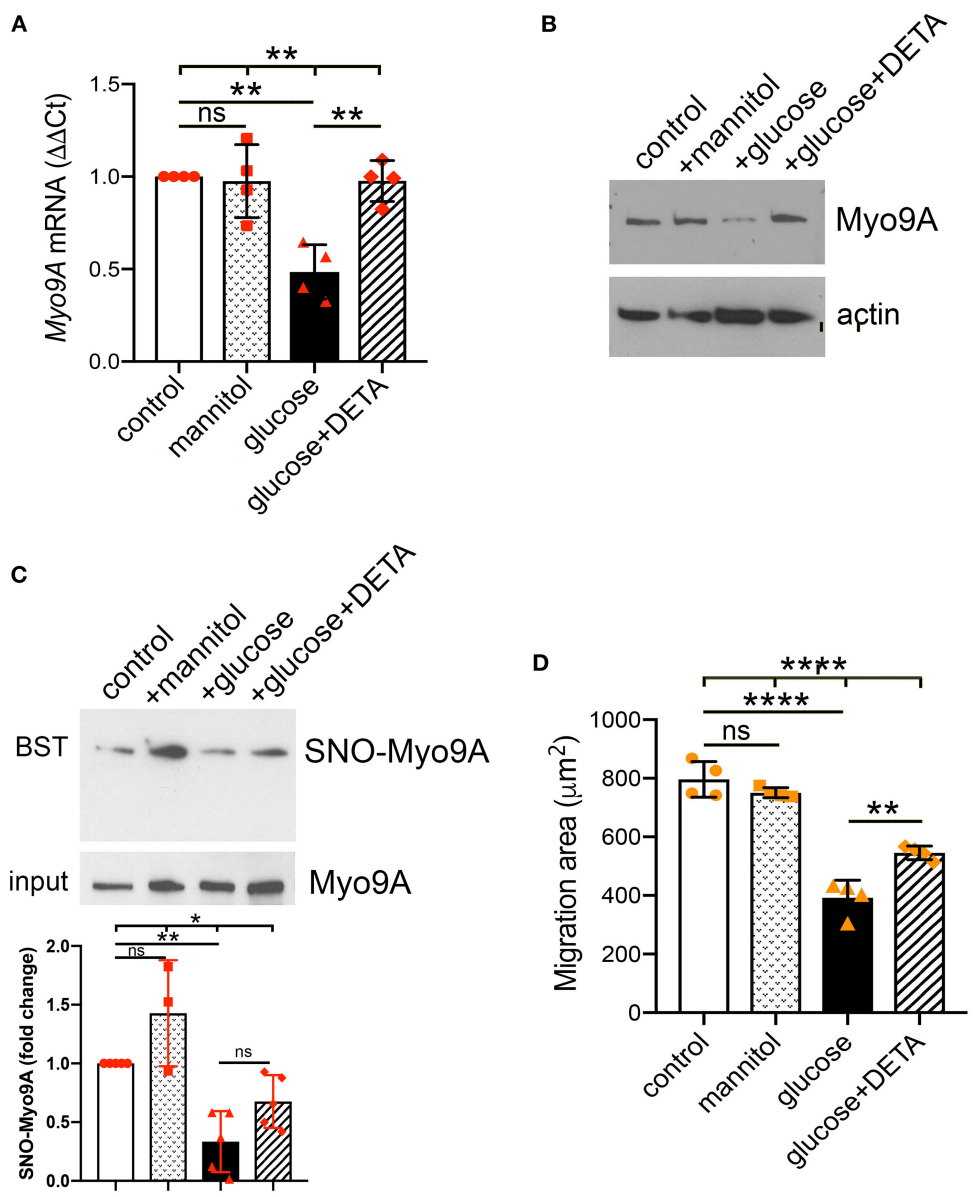
Podocyte Myo9A expression and SNO-Myo9A are regulated by glucose and NO. **(A)** qPCR shows that *Myo9A* mRNA is not affected by mannitol, decreases ~50% in podocytes exposed to high glucose and addition of NO donor prevents *Myo9A* mRNA downregulation, mean ± SD, n = 4 independent experiments; Welch's ANOVA *p* < 0.02, unpaired *t*-test with Welch's correction: n.s. control vs. mannitol, *p* < 0.02 control vs. high glucose, *p* < 0.02 high glucose vs. high glucose + DETA. **(B)** Immunoblots show that Myo9A protein expression is not altered by mannitol, decreases ≥50% in podocytes exposed to high glucose and addition of NO donor prevents Myo9A downregulation. **(C)** BST shows SNO-Myo9A in control podocytes, SNO-Myo9A ~50% decrease in podocytes exposed to high glucose, addition of NO donor partially prevents Myo9A de-nitrosylation. Input shows total Myo9A loading, mean ± SD, *n* = 3–5 independent experiments, Brown-Forsythe ANOVA test, *p* = 0.022, unpaired *t*-test with Welch's correction non-significant (n.s.) control vs. mannitol, **p = 0.0046 control vs. high glucose, *p* = 0.0575 (n.s.) high glucose vs. high glucose + DETA. **(D)** Migration ‘wound' assay shows that podocyte migration is not affected by mannitol, whereas high glucose clearly reduces podocyte migration and addition of NO donor partially prevents this defect, mean ± SD, *n* = 4 independent experiments; Welch's ANOVA *p* < 0.0001, unpaired t-test with Welch's correction non-significant (n.s.) control vs. mannitol, *p* < 0.005 control vs. high glucose, *p* < 0.02 high glucose vs. high glucose + DETA. *p < 0.05, **p < 0.01, ****p < 0.0001.

Since IF revealed decreased podocyte Myo9A protein expression upon exposure to high glucose, we performed qPCR and immunoblotting to quantitate this effect at both mRNA and protein levels. Immortalized differentiated mouse podocytes were exposed to normal glucose, mannitol, high glucose or high glucose + nitric oxide donor (DETA). Podocyte *Myo9A* mRNA and protein decreased significantly upon exposure to high glucose as compared to normal glucose or mannitol (Figures 4A,B). The long acting NO donor DETA abrogated the high glucose-induced defect in *Myo9A* expression (Figures 4A,B).

To examine further the regulation of Myo9A S-nitrosylation we performed biotin-switch test (BST). Consistent with the PLA results (Figures 3C,D), biotin-switch tests (BST) demonstrated that Myo9A S-nitrosylation (SNO-Myo9A) decreases ~50% upon podocyte exposure to high glucose (Figure 4C). Addition of NO donor DETA partially improves the Myo9A S-nitrosylation defect induced by podocyte exposure to high glucose (Figure 4C). We evaluated the effect of high glucose on podocyte function using a migration assay. Upon exposure to high glucose podocyte migration was significantly reduced, as compared to normal glucose or mannitol (Figure 4D). The migration defect was partially abrogated by addition of DETA (Figure 4D).

### S-Nitrosylation of Myo9A Interacting Proteins RhoA and Actin

**Figure 5:**
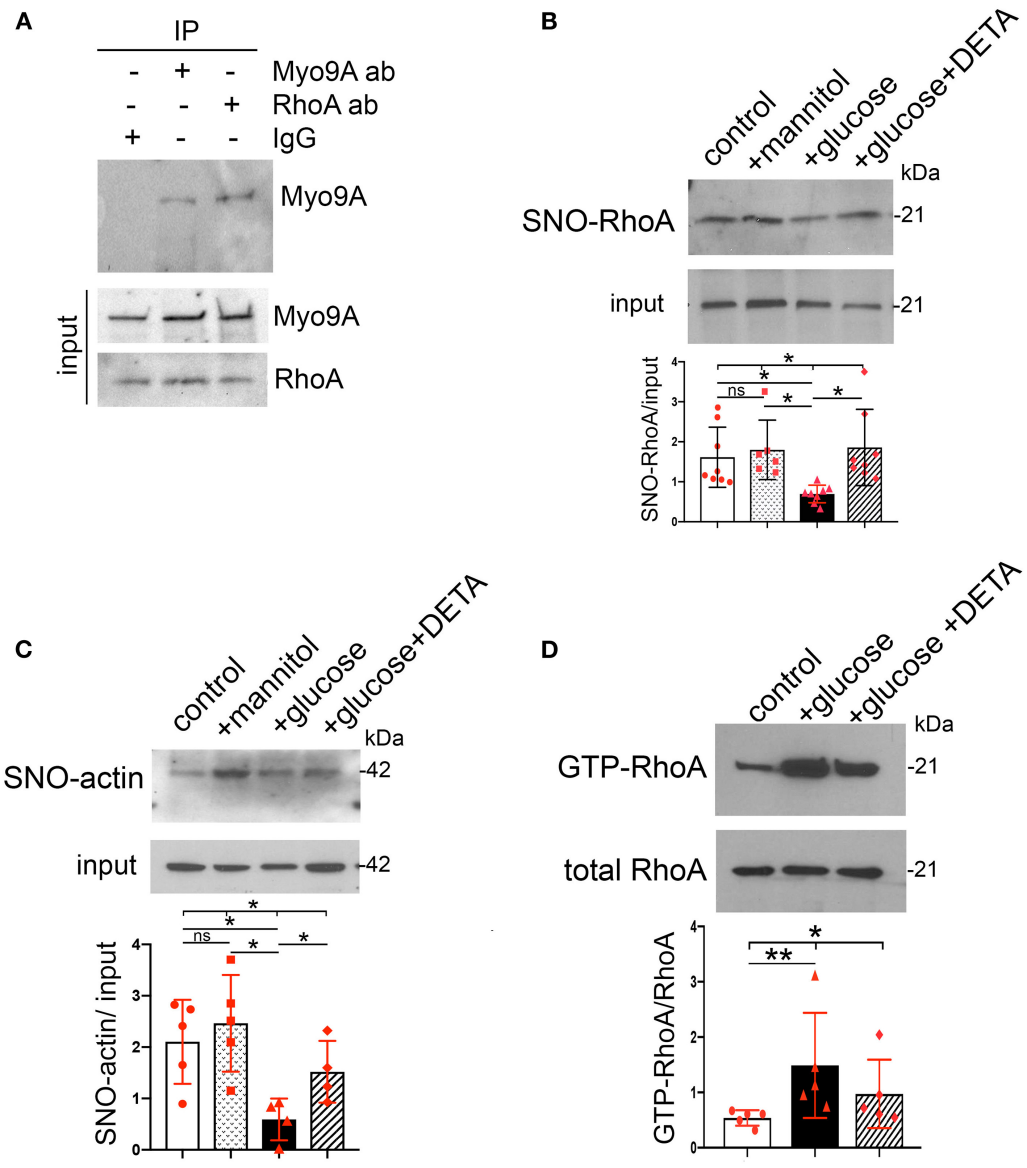
Podocyte SNO-RhoA, SNO-actin and RhoA activity regulation by glucose and NO. **(A)** Immunoprecipitation (IP): Myo9A and RhoA, WB: RhoA and Myo9A demonstrate Myo9A-RhoA interaction in podocytes. **(B)** BST shows SNO-RhoA in normal podocytes, ~50% SNO-RhoA decrease in podocytes exposed to high glucose, addition of NO donor prevents RhoA de-nitrosylation. Input shows total RhoA, mean ± SD, *n* = 6–8 independent experiments, Welch's ANOVA test, *p* = 0.002, unpaired *t*-test with Welch's correction non-significant (n.s.) control vs. mannitol, *p* < 0.01 control vs. high glucose, *p* < 0.01 high glucose vs. high glucose + DETA. **(C)** BST shows SNO-actin >50% decrease induced by high glucose, partially prevented by the NO donor DETA. Input shows actin loading, mean ± SD, *n* = 4–6 independent experiments, Welch's ANOVA test, *p* = 0.013; unpaired *t*-test with Welch's correction n.s. control vs. mannitol, *p* = 0.0011 control vs. high glucose, *p* < 0.05 high glucose vs. high glucose + DETA. **(D)** RhoA activity assay shows that exposure to high glucose increases active GTP-RhoA and addition of NO donor partially prevents activation of RhoA. Total RhoA shows equal input, mean ± SD, *n* = 5 independent experiments, Kruskall-Wallis test, *p* = 0.013; Mann-Whitney test *p* = 0.0079 control vs. high glucose. *p < 0.05 and **p < 0.01.

We assessed whether high glucose and NO regulate S-nitrosylation of Myo9A interacting proteins, RhoA and actin (7, 14). We determined that Myo9A interacts with RhoA in podocytes using immunoprecipitation (Figure 5A). Then, we performed BST to evaluate SNO-RhoA and SNO-actin under the conditions described above. These experiments revealed that both RhoA and actin are S-nitrosylated in control podocytes (Figures 5B,C). Remarkably, high glucose decreased SNO-RhoA, while addition of NO donor DETA abrogated podocyte RhoA de-nitrosylation (Figure 5B). High glucose also induced >50% SNO-actin decrease in podocytes and exposure to NO donor partially prevented this defect (Figure 5C). We measured RhoA activity using a pull down assay (7) and determined that high glucose induces an increase in RhoA activity, which is partially abrogated by NO donor (Figure 5D).

Taken together, our findings indicate that in podocytes high glucose-induced downregulation of SNO-Myo9A is associated with similar decreases in SNO-actin and SNO-RhoA, as well as with increased RhoA activity, all of which are regulated by NO availability.

## Discussion

This study demonstrates that the unconventional myosin Myo9A is S-nitrosylated in normal podocytes and that diabetic milieu downregulates Myo9A expression and S-nitrosylation *in vivo*. Our findings revealed that Myo9A S-nitrosylation is regulated by glucose and nitric oxide availability in cultured podocytes, consistent with *in vivo* findings. Data uncover S-nitrosylation as an integrated signaling between Myo9A and its interacting proteins RhoA and actin that transduces metabolic cues (high glucose + low NO), modifies cytoskeletal effectors (RhoA) function and impacts podocyte behavior.

Using an experimental type 1 diabetes (T1D) mouse model we determined that Myo9A expression in the kidney is decreased in diabetic mice with advanced DKD, while in diabetic mice with mild DKD Myo9A expression is not different from non-diabetic mice (7). Glomerular Myo9A is S-nitrosylated in mice with mild DKD whereas SNO-Myo9A is significantly reduced in mice with advanced DKD. These findings suggest (but do not prove) that downregulation of Myo9A expression and S-nitrosylation are mechanistically involved in the progression or severity of DKD. In this experimental model the development of diabetic nodular glomerulosclerosis is driven by inducible podocyte *VEGF*_164_ overexpression (29). Thus, the observed changes in Myo9A expression and SNO-Myo9A in induced mice with severe DKD could be a direct effect of excess glomerular VEGF-A and not mechanistically contributing to DKD progression. A previous study showed that VEGF-A cell autonomously decreases laminin S-nitrosylation in podocytes (33). Alternatively, hyperglycemia and VEGF_164_-induced NOS uncoupling reduce NO availability (17), which in turn could influence *Myo9A* expression and SNO-Myo9A in diabetic glomeruli and thereby contribute to DKD progression. Results from a DNA array showing >2-fold decrease of *Myo9A* expression in diabetic Zucker rats, a model of type 2 diabetes (T2D) are consistent with the latter possibility (35). Further studies assessing Myo9A role in DKD progression in other experimental mouse models of advanced DKD, e.g., T1D or T2D + *eNOS* KO, are warranted. Here we used podocytes to examine how the diabetic milieu influences Myo9A at the cellular level.

A key finding of this study is that Myo9A is S-nitrosylated in normal podocyte culture conditions and de-nitrosylates in diabetic milieu. Data indicate that this is not due to hyperosmolarity associated with high glucose and it is abrogated by addition of NO donor, demonstrating that SNO-Myo9A is glucose and NO dependent. *Myo9A* expression is also glucose and NO dependent, raising the intriguing possibility that *Myo9A* regulation is both transcriptional and post-translational in podocytes. High glucose-induced Myo9A downregulation and de-nitrosylation were associated with decreased podocyte migration, which was partially abrogated by a NO donor. Although this abnormal podocyte behavior in the diabetic milieu could be mediated via multiple pathways, it is remarkably similar to that reported in *Myo9A* knockdown podocytes (7), suggesting that Myo9A dysregulation is involved.

Myo9A binds actin at one of the two actin-binding sites in loop 2 of the catalytic domain forming crosslinks that bridge across actin filaments in parallel polarity at 36 nm regular intervals matching the actin helical repeat, thereby bundling actin filaments to form ordered networks (36). Experimental conditions such as calcium-calmodulin, ATP and redox status influence Myo9A actin crosslinking activity *in vitro* (36). However, it is presently unknown whether SNO-Myo9A is required for actin crosslinking *in vivo*.

We report for the first time SNO-actin in normal podocytes, which is regulated by high glucose and NO dependent alike SNO-Myo9A. In physiological conditions all actin isoforms are S-nitrosylated on Cys374 and probably on additional Cys residues (37). Because actin is abundantly expressed and largely S-nitrosylated in most cells, it has been proposed that actin serves as a cell SNO-thiol reservoir that trans-nitrosylates with GSH-nitroso-glutathione (GSNO) (24, 26). S-nitrosylation affects actin polymerization and its interaction with proteins that are relevant for actin dynamics, including VASP, cofilin1, profilin and α-actinin (37).

An important finding of this work is that SNO-RhoA occurs in normal podocytes and inversely relates with RhoA activity. Myo9A interacts directly with RhoA through its tail RhoGAP domain (14, 28). Upon binding, Myo9A dephosphorylates RhoA GTPase rendering it inactive (14). We recently reported that *Myo9A* haploinsufficiency increases RhoA activity in kidneys and podocytes, consistent with loss of RhoGTPase function (7). Here we show that SNO-RhoA is regulated by high glucose and NO dependent, i.e., inversely related to high glucose and positively related to NO availability, and that RhoA activity is inversely related to SNO-RhoA in podocytes. Our results are consistent with a report showing that endothelial cell RhoA S-nitrosylation occurs in physiological conditions, is NO dependent and inhibited by increased intracellular Ca^+2^, while RhoA activity is inversely correlated to SNO-RhoA (38). It is well-established that RhoA activity is elevated in T1D and T2D experimental models and that high glucose increases RhoA activity in endothelial cells (39), mesangial cells (40) and podocytes (41). Collectively, these findings suggest that RhoA de-nitrosylation induced by the diabetic milieu may mediate RhoA activation in all three glomerular cell types.

**Figure 6:**
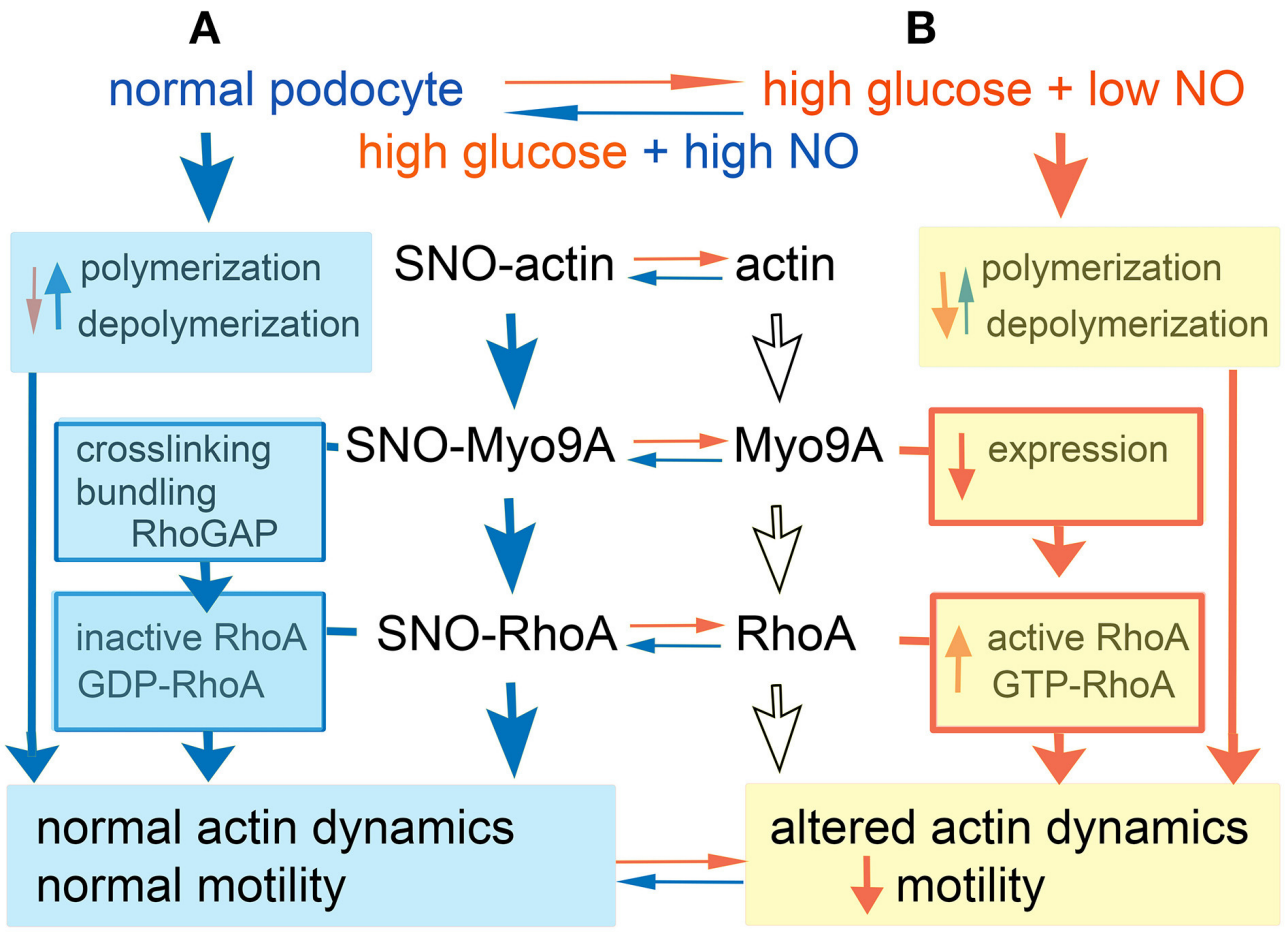
Model. **(A)** In normal podocyte culture conditions actin, Myo9A and RhoA are S-nitrosylated (SNO), whereby F actin polymerization predominates, Myo9A crosslinks and bundles actin and inactivates RhoA leading to normal actin dynamics and podocyte motility. **(B)** In high glucose + low NO conditions podocyte SNO-Myo9A, SNO-RhoA and SNO-actin decrease significantly (de-nitrosylate), actin depolymerization may predominate, Myo9A expression decreases and RhoA activity increases, together resulting in altered actin dynamics and reduced podocyte motility. Most of these changes are partially reversible by addition of NO donor.

Our novel findings of podocyte Myo9A, actin and RhoA S-nitrosylation, which are regulated similarly, suggest that these post-translational modifications are linked. SNO transnitrosylation, i.e., the transfer of NO^−^ between Cys residues, occurs between interacting proteins or those closely adjacent within a cell compartment or microdomain, and its specificity is spatially determined (19, 42, 43). We have previously described Myo9A-actin interaction in podocytes (7). Here we report for the first time Myo9A-RhoA interaction in podocytes. We propose a model of transnitrosylation cascade involving Myo9A, RhoA and actin, three interacting proteins that are critical for podocyte cytoskeleton homeostasis (Figure 6). In this model, the three proteins are S-nitrosylated in control conditions (A) and Myo9A interacts physically and functionally with actin through its catalytic domain that hydrolyses ATP, crosslinks and bundles actin, as well as with RhoA via the tail RhoGAP domain that de-phosphorylates and inactivates the RhoA GTPase (14, 36). In the diabetic milieu (B), high glucose and low NO decrease SNO-Myo9A, SNO-actin and SNO-RhoA leading to increased RhoA activity and abnormal actin dynamics, which alter podocyte function, as assessed by decreased migration. Remarkably, these changes are at least partially reversible. SNO-actin changes upon high-glucose exposure are consistent with the hypothesis that it acts as a SNO reservoir (26). Alternatively, S-nitrosylation of actin isoforms may be regulated differently (24, 44). We speculate that SNO-RhoA may also be regulated by TRPC6-mediated increases in iCa^+2^, known to be stimulated by AngII and VEGF-A and to mediate RhoA activation in the diabetic milieu (45–47). Akin to SNO inhibition of PKM2 (48–50), the reversible inhibitory S-nitrosylation of RhoA described herein may provide a novel mode of regulation amenable to therapeutic intervention in DKD.

Further studies are needed to provide detailed insight on the proposed model. For example, it is critical to elucidate whether SNO-MyoA is required for Myo9A's actin crosslinking activity and RhoGAP function and to determine how does SNO-actin influence the balance of actin polymerization-depolymerization dynamics in podocytes. Actin is also oxidized on Met44 and Met47 by MICAL, a flavo-oxygenase expressed in the kidney and in podocytes (51, 52) that leads to F-actin disassembly (52, 53). It is not known if oxidation of actin Met residues is regulated by glucose or NO dependent. Characterization of Myo9A post-translational modifications is limited (14). Identification of Myo9A Cys residues that undergo S-nitrosylation has been elusive as yet, precluding definitive experiments testing our model. Limitations of this study include not examining S-nitrosylation of Myo9A and its interacting partners in biopsy samples from DKD patients, in proximal tubular cells, glomerular endothelial and mesangial cells, known to be involved in DKD progression, nor in additional experimental T1D and T2D models. Further *in vivo* studies are needed to ascertain whether decreased SNO-RhoA and SNO-actin contribute to DKD progression and to evaluate the effect of NO donors on S-nitrosylation of Myo9A, RhoA and actin.

In summary, this work shows that Myo9A, RhoA and actin are S-nitrosylated in normal podocytes and that diabetic milieu induces Myo9A, actin and RhoA de-nitrosylation, resulting in increased RhoA activity and impaired podocyte migration, which proved to be partially reversible, and therefore potentially targetable. Collectively, our findings uncover S-nitrosylation of Myo9A, actin and RhoA as an integrated signaling crosstalk that reversibly transduces metabolic cues to regulate actin dynamics and podocyte motility.

## Data Availability Statement

The raw data supporting the conclusions of this article will be made available by the authors, without undue reservation.

## Ethics Statement

The animal study was reviewed and approved by Institutional Animal Care and Use Committee at the Yale University School of Medicine.

## Author Contributions

QL and DV performed experiments, analyzed data, and contributed to manuscript writing. AT designed and supervised experiments, analyzed data, and wrote the manuscript. All authors contributed to the article and approved the submitted version.

## Conflict of Interest

The authors declare that the research was conducted in the absence of any commercial or financial relationships that could be construed as a potential conflict of interest.
